# Silencing IL12p35 Promotes Angiotensin II-Mediated Abdominal Aortic Aneurysm through Activating the STAT4 Pathway

**DOI:** 10.1155/2021/9450843

**Published:** 2021-07-27

**Authors:** Lanlan Wang, Chengyun Hu, Yongfei Dong, Feibiao Dai, Yongxia Xu, Yumeng Dai, Lijie Shao, Defa Zhu

**Affiliations:** ^1^Department of Geriatric Endocrinology, The First Affiliated Hospital of Anhui Medical University, Hefei 230032, China; ^2^Department of Geriatric Endocrinology, The First Affiliated Hospital of USTC, Division of Life Sciences and Medicine, University of Science and Technology of China, Hefei, Anhui 230001, China; ^3^Department of Anesthesiology, The First Affiliated Hospital of USTC, Division of Life Sciences and Medicine, University of Science and Technology of China, Hefei, Anhui 230001, China; ^4^Department of Neurosurgery, The First Affiliated Hospital of USTC, Division of Life Sciences and Medicine, University of Science and Technology of China, Hefei, Anhui 230001, China

## Abstract

*Background and Purpose*. Abdominal aortic aneurysm (AAA) is a chronic inflammatory disorder and the important causes of death among men over the age of 65 years. Interleukin-12p35 (IL12p35) is an inflammatory cytokine that participates in a variety of inflammatory diseases. However, the role of IL12p35 in the formation and development of AAA is still unknown. *Experimental Approach*. Male apolipoprotein E-deficient (Apoe^−/−^) mice were generated and infused with 1.44 mg/kg angiotensin II (Ang II) per day. We found that IL12p35 expression was noticeably increased in the murine AAA aorta and isolated aortic smooth muscle cells (SMCs) after Ang II stimulation. IL12p35 silencing promoted Ang II-induced AAA formation and rupture in Apoe^−/−^ mice. IL12p35 silencing markedly increased the expression of inflammatory cytokines, including IL-1*β*, IL-6, and tumor necrosis factor-*α* (TNF-*α*), in both the serum and AAA aorta. Additionally, IL12p35 silencing exacerbated SMC apoptosis in Apoe^−/−^ mice after Ang II infusion. IL12p35 silencing significantly increased signal transducer and activator of transcription (STAT) 4 phosphorylation levels in AAA mice, and STAT4 knockdown abolished the IL12p35-mediated proinflammatory response and SMC apoptosis. *Interpretation*. Silencing IL12p35 promotes AAA formation by activating the STAT4 pathway, and IL12p35 may serve as a novel and promising therapeutic target for AAA treatment.

## 1. Introduction

Abdominal aortic aneurysm (AAA) is a chronic inflammatory disorder and one of the important causes of death among men over the age of 65 years [1, 2]. AAA is defined as localized enlargement of the abdominal aorta to a diameter of at least 50% greater than its normal size or an aorta that measures greater than 30 mm in diameter [3]. Epidemiological data have shown that the incidence rate of AAA is approximately 1.4%~12.4% and directly causes 150,000~200,000 deaths each year worldwide due to AAA rupture [4, 5]. In addition, the mortality rate of AAA exceeds 80% after rupture despite surgical advancements [3, 6]. Effective pharmacotherapies for halting the growth and rupture of AAA or delaying the need for surgical repair are currently lacking.

Although the pathogenesis of AAA is complex, degeneration of the aortic media has been proven to be a crucial hallmark of AAA pathology [7, 8]. High numbers of smooth muscle cells (SMCs), elastic fiber fragmentation, and excessive collagen and proteoglycan accumulation are the main characteristics of medial degeneration and are key factors in AAA dilation and rupture [9, 10]. In addition, the inflammatory response, oxidative stress, SMC apoptosis, and proteolytic degradation of aortic wall connective tissue have all been associated with the development of AAA [11, 12]. Accordingly, the pharmacological regulation of these processes represents a promising strategy to control the progression of AAA.

Interleukin-12 (IL-12) is a pleiotropic cytokine with multiple immunomodulatory effects and is composed of p35 and p40 subunits [13, 14]. The biological effect mediated by IL-12 is closely associated with signal transducer and activator of transcription (STAT) signaling, specifically STAT4 [15, 16]. IL-12 has been identified as an important inflammation-related cytokine involved in the regulation of several cardiovascular diseases, including myocardial infarction [17], atherosclerosis [18], and hypertension [19]. Recent studies have demonstrated that IL12p40 deficiency accelerates pathological matrix remodeling and promotes the development of AAA [20, 21]. However, the direct role of IL12p35 in the development of AAA had not yet been determined. In this study, we identified the role of IL12p35 in the development of AAA and attempted to elucidate the possible mechanism.

## 2. Materials and Methods

### 2.1. Animals and Animal Model

Nine- to eleven-week-old male apolipoprotein E-deficient (Apoe^−/−^) mice generated on a C57BL/6J background were purchased from Hunan SJA Laboratory Co., Ltd. (Hunan, China) and housed under pathogen-free conditions pm a 12-h light/dark cycle. All animal protocols were approved by the Animal Care and Use Committee of Anhui Medical University.

To silence IL12p35 in vivo, adeno-associated viruses (AAVs) carrying IL12p35 shRNA (AAV-shIL12p35) and scramble RNA (AAV-shRNA) were constructed and generated by Shandong Vigenebio Co., Ltd. (Shandong, China). The mice were randomly assigned to two groups and received injections of AAV-shIL12p35 (1 × 1011 vector genomes) and AAV-shRNA (1 × 1011 vector genomes) via the tail vein. After 4 weeks, all mice were implanted with osmotic pumps and received a continuous subcutaneous infusion of angiotensin II (Ang II) or saline as described previously [22]. In brief, the animals were anesthetized and then placed in a prone position on a heating pad. Next, an Alzet osmotic pump (Model 2004, USA) loaded with Ang II (1.44 mg/kg per day, Calbiochem, USA) or saline was implanted subcutaneously in the nape of the neck. After 4 weeks, murine aortas and blood samples were collected to perform further analyses.

### 2.2. Ultrasonic Imaging

Upon completion of Ang II infusion, the mice were anesthetized, and ultrasound analysis of the abdominal aorta was performed using a Vevo 2100 System (Visual Sonics, Canada) equipped with a linear array ultrasound transducer. The maximum aortic diameters of the mice were measured three times by an investigator who was blinded to the group information.

### 2.3. Aneurysm Quantification

For aneurysm analysis, the aortas were isolated, and the periadventitial tissue was removed. The maximal aortic diameter was measured using the ImageJ software (NIH, USA). Aneurysm formation was identified as an enlargement >50% of the diameter compared with that of mice without Ang II infusion as described previously [23]. In addition, the incidence of aneurysms and the survival rate were monitored and calculated. Two colleagues who were blinded to the group information carried out the aneurysm quantification analysis.

### 2.4. Histological Analysis

Murine aortas were fixed with 4% paraformaldehyde and embedded in paraffin. Serial sections (7 *μ*m) were created and analyzed as previously described [24]. Hematoxylin and eosin (H&E) staining was performed to assess aortic morphology. Elastin staining was performed to assess elastin degradation. In addition, a commercially available terminal deoxynucleotidyl transferase dUTP nick-end labeling (TUNEL) detection kit was used to assess SMC apoptosis.

### 2.5. Cell Culture and Treatments

Aortic SMCs were isolated from Apoe^−/−^ mice and cultured in DMEM supplemented with 10% fetal bovine serum (FBS) as previously described [25]. Then, SMCs were transfected with si-IL12p35 in the presence or absence of Ang II (1 *μ*mol/L) for 24 h. To knockdown STAT4, SMCs were transfected with si-STAT4 using Lipofectamine 2000 (Thermo Fisher Scientific, USA) in accordance with the manufacturer's instructions.

### 2.6. Quantitative Real-Time PCR (qRT-PCR)

Total RNA was extracted from aortic tissues or cultured aortic SMCs using an RNeasy kit (Qiagen, Germany), and 2-*μ*g aliquots of total RNA were used for first-strand cDNA synthesis using reverse transcriptase (TaKaRa, Japan) according to the manufacturer's recommendation. qRT-PCR was performed using a SYBR Green RT-PCR kit (TaKaRa, Japan), and *β*-actin was used to normalize gene expression. The primer sequences are shown in Table 1.

### 2.7. Western Blot Analysis

Total protein was extracted from abdominal aortic tissues or cultured aortic SMCs using RIPA buffer according to the manufacturer's instructions. Then, the protein samples were loaded onto SDS-PAGE gels and electrophoretically transferred to an Immobilon-P membrane. The membranes were probed with primary antibodies at 4°C overnight, after which the membranes were incubated with secondary antibodies. Finally, the membranes were visualized, and the density of each target protein band was normalized to the corresponding density of the *β*-actin band.

### 2.8. Enzyme-Linked Immunosorbent Assay (ELISA)

Mouse blood was collected and separated by centrifugation to prepare serum. Then, the concentrations of IL-1*β*, IL-6, and tumor necrosis factor- (TNF-) *α* in the serum samples were measured using ELISA kits (Arigo Biolaboratories, China) according to the manufacturer's protocols.

### 2.9. Statistical Analysis

The data are presented as the means ± standard deviation (SD). Continuous data were examined by the Shapiro-Wilk test for normality. After confirming variance equality between different groups, statistical comparisons between 2 groups were performed using Student's *t*-tests, whereas significant comparisons between multiple groups were performed using one-way analysis of variance (ANOVA)with post hoc Bonferroni's tests. For variables with a nonnormal distribution, the nonparametric Mann–Whitney *U* test was performed to assess the differences between the 2 groups. In addition, the AAA incidence in each group was compared using Fisher's exact test. Kaplan-Meier analysis was performed to assess the survival rate using the log-rank test. *P* values less than 0.05 were considered significant.

## 3. Results

### 3.1. IL12p35 Expression Is Increased in Murine AAA Aorta and Isolated Aortic SMCs

**Figure 1:**
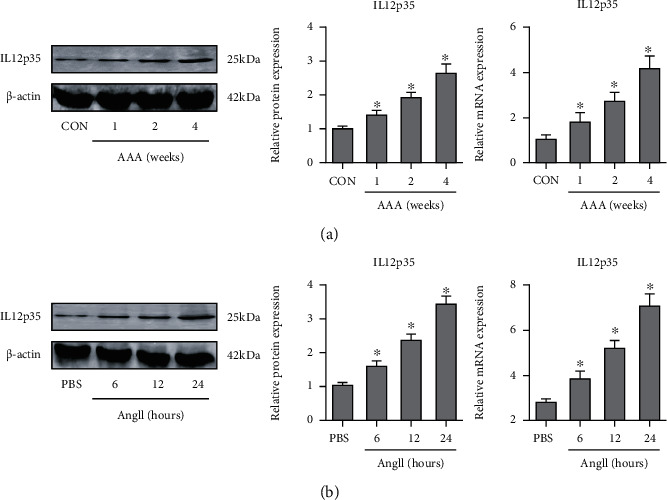
IL12p35 expression is increased in the murine AAA aorta and isolated aortic SMCs. (a) Western blots and PCR analysis of IL12p35 expression in murine aortic samples (*n* = 5 per group). (b) Western blots and PCR analysis of IL12p35 expression in isolated aortic SMCs (*n* = 5 per group). ^∗^*P* < 0.05, compared with the control or PBS group.

We first examined IL12p35 expression in the murine AAA aorta and aortic SMCs. IL12p35 protein levels were progressively elevated in the aorta from 1 to 4 weeks after Ang II infusion in the experimental AAA model mice compared with control mice (1.4-, 1.9-, and 2.6-fold at 1, 2, and 4 weeks, respectively) (Figure 1(a)). In addition, IL12p35 mRNA levels gradually increased in murine AAA aorta (Figure 1(a)). Consistent with this finding, increased IL12p35 levels were also detected in isolated aortic SMCs after Ang II stimulation (Figure 1(b)).

### 3.2. IL12p35 Silencing Promotes Ang II-Induced AAA Formation and Rupture

**Figure 2:**
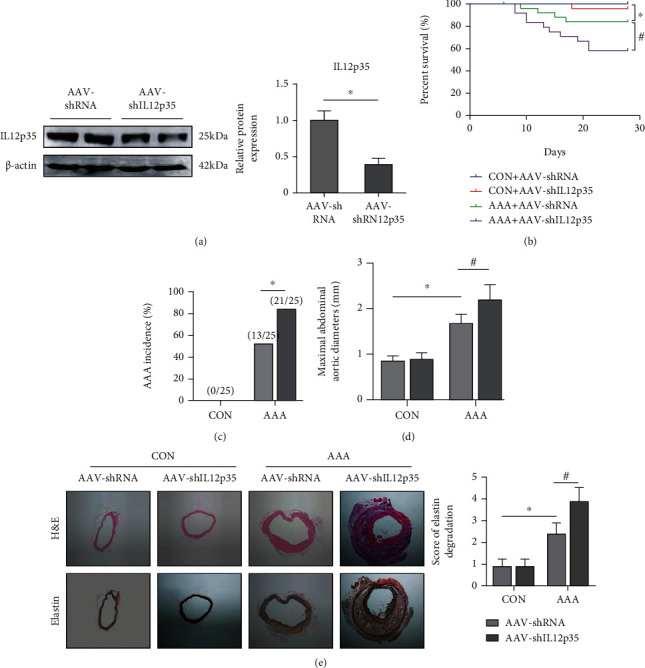
IL12p35 silencing promotes Ang II-induced AAA formation and rupture. (a) Western blot analysis of IL12p35 expression after AAV-shIL12p35 injection in mice (*n* = 5 per group). (b) The survival curves of mice in the four groups (*n* = 25 per group). (c) The rupture rates of mice in the four groups (*n* = 25 per group). (d) Maximal abdominal aortic diameters of mice in the four groups (*n* = 10 per group). (e) Representative H&E and elastin staining in the four groups of mice (*n* = 8 per group). ^∗^*P* < 0.05 compared with the control group; ^#^*P* < 0.05 compared with the AAA group.

To investigate the functional role of IL12p35 in AAA, the mice were given injections of AAV-shIL12p35 via the tail vein to silence IL12p35 in vivo (Figure 2(a)). After 4 weeks of chronic Ang II infusion, aortic rupture-induced mortality was higher in the AAV-shIL12p35 group than in the AAV-shRNA group (40.0% versus 16.0%) (Figure 2(b)). The AAA incidence was higher in the AAV-shIL12p35 group than in the AAV-shRNA group after Ang II infusion (84.0% versus 52.0%) (Figure 2(c)). In addition, IL12p35 silencing also increased the maximal aortic diameter and elastin degradation score after Ang II infusion (Figures 2(d) and 2(e)).

### 3.3. IL12p35 Silencing Exacerbates the Inflammatory Response after Ang II Infusion

**Figure 3:**
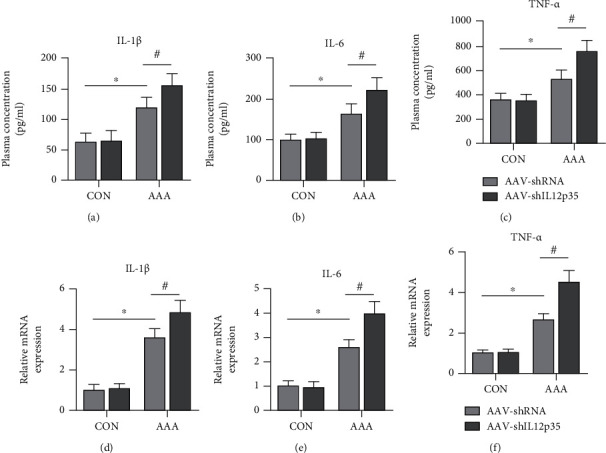
IL12p35 silencing exacerbates the inflammatory response after Ang II infusion. (a–c) ELISA analysis of serum IL-1*β*, IL-6, and TNF-*α* levels of mice in the four groups (*n* = 8 per group). (d–f) PCR analysis of IL-1*β*, IL-6, and TNF-*α* mRNA expression in murine aortic samples (*n* = 8 per group). ^∗^*P* < 0.05 compared with the control group; ^#^*P* < 0.05 compared with the AAA group.

Compared with those in the control group, serum concentrations of inflammatory cytokines, including IL-1*β*, IL-6, and TNF-*α*, in the AAA group were significantly increased, while IL12p35 silencing further increased the serum levels of these cytokines (Figures 3(a)–3(c)). Furthermore, IL12p35 silencing also increased the mRNA expression of IL-1*β*, IL-6, and TNF-*α* in aortic tissues after Ang II infusion (Figures 3(d)–3(f)).

### 3.4. IL12p35 Silencing Exacerbates SMC Apoptosis after Ang II Infusion

**Figure 4:**
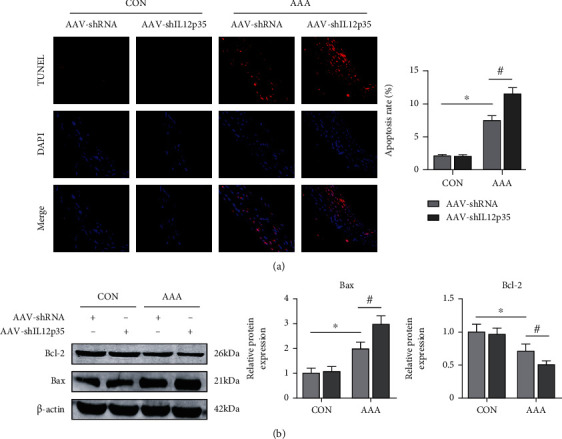
IL12p35 silencing exacerbates SMC apoptosis after Ang II infusion. (a) Representative TUNEL staining in the four groups of mice (*n* = 8 per group). (b) Western blot analysis of the protein levels of Bcl-2 and Bax in the aortas of the four groups (*n* = 5 per group). ^∗^*P* < 0.05 compared with the control group; ^#^*P* < 0.05 compared with the AAA group.

The TUNEL staining results showed that IL12p35 silencing further increased SMC apoptosis after Ang II infusion (Figure 4(a)). The Western blot results also showed that the expression of Bcl-2 was significantly decreased and that the expression of Bax was significantly increased in the AAA group compared with the control group (Figure 4(b)). However, IL12p35 silencing further reduced Bcl-2 expression and increased Bax expression after Ang II infusion (Figure 4(b)).

### 3.5. IL12p35 Silencing Activates the STAT4 Signaling Pathway

**Figure 5:**
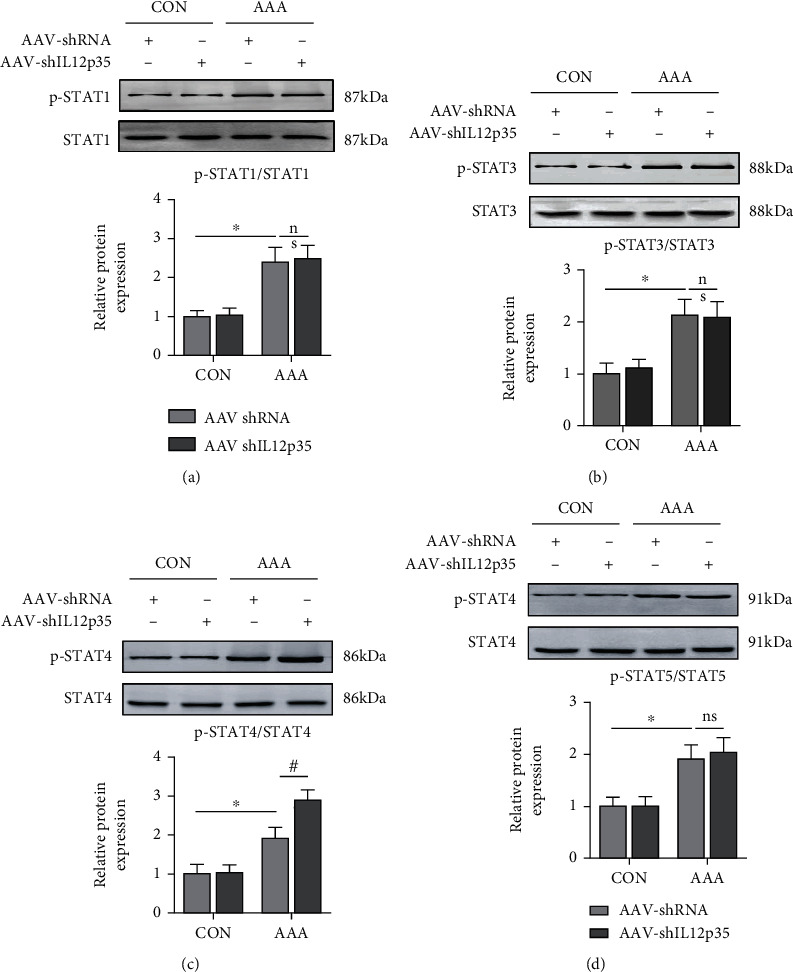
IL12p35 silencing activates the STAT4 signaling pathway. (a–d) Western blot analysis of the protein levels of p-STAT1, STAT1, p-STAT3, STAT3, p-STAT4, STAT4, p-STAT5, and STAT5 in the aortas of the four groups (*n* = 5 per group). ^∗^*P* < 0.05 compared with the control group; ^#^*P* < 0.05 compared with the AAA group.

We further investigated the molecular mechanisms by which IL12p35 affected the development of AAA. The results showed that Ang II infusion significantly increased the phosphorylation of STAT1 (2.3-fold), STAT3 (2.1-fold), STAT4 (1.9-fold), and STAT5 (1.9-fold) (Figures 5(a)–5(d)). In addition, IL12p35 silencing further increased the expression of p-STAT4 (2.9-fold) but did not affect the expression of p-STAT1, p-STAT3, or p-STAT5 (Figure 5(a)–5(d)).

### 3.6. IL12p35 Silencing Exacerbates the Ang II-Induced Inflammatory Response and SMC Apoptosis

**Figure 6:**
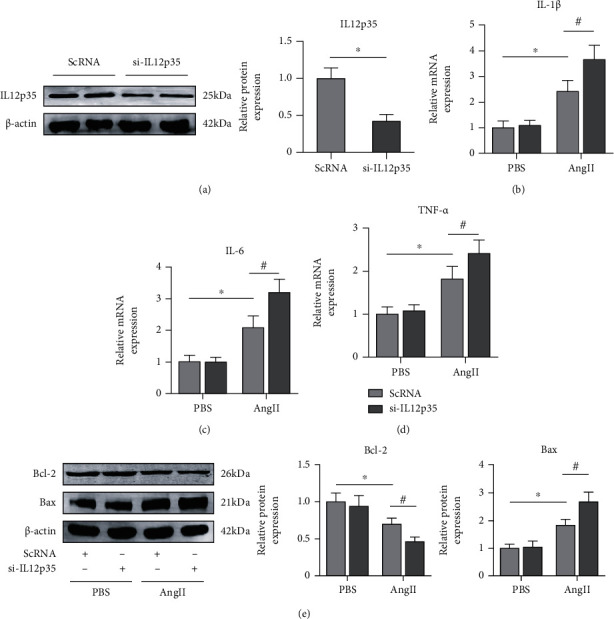
IL12p35 silencing exacerbates the Ang II-induced inflammatory response and SMC apoptosis. (a) Western blot analysis of IL12p35 expression after transfection with si-IL12p35 (*n* = 5 per group). (b–d) PCR analysis of IL-1*β*, IL-6, and TNF-*α* mRNA expression in the four groups (*n* = 6 per group). (e) Western blot analysis of the protein levels of Bcl-2 and Bax in the four groups (*n* = 5 per group). ^∗^*P* < 0.05 compared with the PBS group; ^#^*P* < 0.05 compared with the Ang II group.

To investigate the functional role of IL12p35 in AAA, SMCs were transfected with si-IL12p35 to silence IL12p35 (Figure 6(a)). The results showed that Ang II stimulation significantly increased the mRNA expression of IL-1*β*, IL-6, and TNF-*α*, while IL12p35 silencing further increased the expression of those inflammatory markers (Figures 6(b)–6(d)). In addition, IL12p35 silencing further significantly enhanced Ang II-induced SMC apoptosis by decreasing Bcl-2 expression and increasing Bax expression (Figure 6(e)).

### 3.7. STAT4 Silencing Abolishes IL12p35-Mediated Inflammatory Responses and SMC Apoptosis

**Figure 7:**
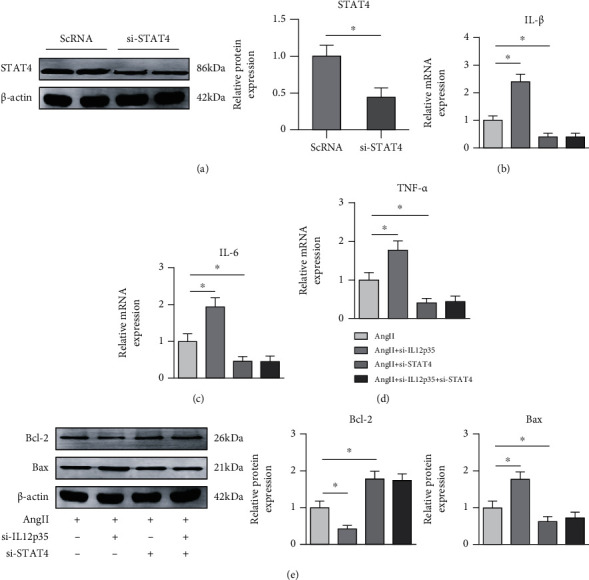
STAT4 silencing abolishes IL12p35-mediated inflammatory responses and SMC apoptosis. (a) Western blot analysis of STAT4 expression after transfection with si-STAT4 (*n* = 4 per group). (b–d) PCR analysis of IL-1*β*, IL-6, and TNF-*α* mRNA expression in the four groups (*n* = 6 per group). (e) Western blot analysis of the protein levels of Bcl-2 and Bax in the four groups (*n* = 4 per group). ^∗^*P* < 0.05 compared with the Ang II group.

To further confirm the effect of the STAT4 signaling pathway on IL12p35-mediated inflammatory responses and SMC apoptosis, cells were transfected with si-STAT4 to silence STAT4 expression in vitro (Figure 7(a)). The results showed that STAT4 silencing abolished the IL12p35-mediated proinflammatory effects by decreasing IL-1*β*, IL-6, and TNF-*α* mRNA expression (Figures 7(b)–7(d)). In addition, STAT4 silencing attenuated IL12p35-mediated SMC apoptosis by increasing Bcl-2 expression and decreasing Bax expression (Figure 7(e)).

## 4. Discussion

In the present study, we identified the role of IL12p35 in exacerbating the growth and rupture of AAA. We provided direct evidence that IL12p35 expression was noticeably increased in the murine AAA aorta and aortic SMCs after Ang II stimulation. In addition, IL12p35 silencing promoted Ang II-induced AAA formation and rupture in Apoe^−/−^ mice by activating the inflammatory response and SMC apoptosis. Moreover, the deterioration caused by IL12p35 silencing was associated with STAT4 activation. These findings suggest that IL12p35 could be a promising target to control the progression of AAA.

In our study, the murine AAA model was induced by subcutaneous infusion of Ang II into Apoe^−/−^ mice for 4 weeks. This model mimics some of the pathological features of human AAA, such as aortic rupture, inflammation, and important risk factors, and it is also technically appropriate for operations [26, 27]. Based on these advantages, this has become one of the main methods used to induce AAA in mice [28, 29]. In the present study, we successfully established an AAA model in Apoe^−/−^ mice, and the results showed that IL12p35 expression was noticeably increased in the murine AAA aorta and isolated aortic SMCs after Ang II stimulation. To further investigate the role of IL12p35 in the development of AAA, we utilized AAV transfection to silence IL12p35 expression in AAA mice, and the results showed that silencing IL12p35 increased AAA formation and rupture-induced mortality. In addition, IL12p35 silencing also increased the maximal aortic diameter and elastin degradation score after Ang II infusion.

Although the pathogenesis of AAA is complex, inflammatory responses have been proven to be crucial in the formation of AAA [30, 31]. Many clinical studies and animal experiments have confirmed that extensive inflammatory cell infiltration occurs in both the media and adventitia and is associated with increased aneurysm diameter [32, 33]. In addition, these infiltrating cells also secrete various inflammatory cytokines, such as IL-1*β*[34], IL-6 [35], and TNF-*α* [36], which further drive inflammatory responses and ultimately lead to AAA rupture. Studies have suggested that reducing the activation of these proinflammatory cytokines could inhibit the formation and progression of AAA [34–37]. Thus, it is clear that therapeutic drugs targeting the inflammatory response can be very effective in improving AAA.

Current literatures have described that IL12p35 is an important inflammation-related cytokines involved in the regulation of several inflammation-related diseases [38]. Thus, we investigated the effect of IL12p35 silencing on the inflammatory response in the development of AAA. In line with data from previous research, our results showed that serum and aortic levels of IL-1*β*, IL-6, and TNF-*α* in the AAA group were significantly increased compared with those in the control group, while IL12p35 silencing further increased the expression of these cytokines. In addition, IL12p35 silencing also increased the IL-1*β*, IL-6, and TNF-*α* levels in isolated aortic SMCs after Ang II stimulation. These results suggested that IL12p35 silencing promotes Ang II-induced AAA formation by exacerbating the inflammatory response.

SMCs are one of the most important cellular components of the aortic wall and play a pivotal role in maintaining normal aortic structure and homeostasis [39]. SMCs can synthesize and secrete extracellular matrix proteins and induce aortic wall remodeling in response to increased hemodynamic pressure [39, 40]. A large number of studies have shown that AAA formation is associated with the loss of SMCs due to enhanced apoptosis [41]. In addition, the inflammatory response has been shown to be responsible for SMC apoptosis [42]. Thus, we examined whether IL12p35 affected SMC apoptosis during the development of AAA. The results indicated that the expression of Bax was upregulated and that the expression of Bcl-2 was lower in the AAA group than in the control group. The TUNEL staining results also showed that the apoptosis rates of SMCs in the AAA group were significantly increased compared with those in the control group, and these effects were further augmented by IL12p35 silencing. In addition, these results further confirmed the in vitro results, indicating that the effect of IL12p35 silencing was associated with exacerbation of SMC apoptosis.

Signaling through the STAT pathway is an important regulator of vascular diseases, including hypertension [43], atherosclerosis [44], pulmonary hypertension [45], and AAA [46]. An accumulating body of evidence has indicated that STAT signaling is activated during the early stage of AAA and mediates the inflammatory response, oxidative stress, and SMC apoptosis [47, 48]. Extensive data have also demonstrated that the biological effect mediated by IL-12 is closely associated with STAT signaling, including STAT1, STAT3, STAT4, and STAT5 [49, 50]. Thus, we examined whether IL12p35 affected specific STAT transcription factors during the development of AAA. In the present study, we found that IL12p35 silencing did not affect STAT1, STAT3, or STAT5 phosphorylation but induced STAT4 phosphorylation during the development of AAA. Moreover, STAT4 knockdown nearly abolished the IL12p35 silencing-induced increase in the inflammatory response and SMC apoptosis after Ang II stimulation. Taken together, these results showed that STAT4 activation is critical for IL12p35 silencing-mediated inflammatory responses and SMC apoptosis, which may contribute to AAA formation and rupture.

In summary, our work provides preliminary evidence clarifying the role of IL12p35 in the development of AAA. Silencing of IL12p35 increased AAA formation and rupture in Apoe^−/−^ mice by activating the STAT4 pathway. Our findings suggest that IL12p35 may serve as a novel and promising therapeutic target for AAA treatment.

## Figures and Tables

**Table 1:** Primers used for qRT-PCR.

Gene	Direction	Primer
IL12p35	Forward	AGTTTGGCCAGGGTCATTCC
	Reverse	TCTCTGGCCGTCTTCACCAT
IL-1*β*	Forward	GCAACTGTTCCTGAACTCAACT
	Reverse	ATCTTTTGGGGTCCGTCAACT
IL-6	Forward	CTGCAAGAGACTTCCATCCAG
	Reverse	AGTGGTATAGACAGGTCTGTTGG
TNF-*α*	Forward	CCCTCACACTCAGATCATCTTCT
	Reverse	GCTACGACGTGGGCTACAG
*β*-Actin	Forward	TATTGGCAACGAGCGGTTCC
	Reverse	GGCATAGAGGTCTTTACGGATGT

## Data Availability

The datasets generated and/or analyzed during the current study are available from the corresponding author on reasonable request in compliance with ethical standards.
